# Mobile App Design, Development, and Publication for Adverse Drug Reaction Assessments of Causality, Severity, and Preventability

**DOI:** 10.2196/mhealth.6261

**Published:** 2017-05-30

**Authors:** Muslimah Ithnin, Mohd Dzulkhairi Mohd Rani, Zuraidah Abd Latif, Paveethra Kani, Asmalita Syaiful, Khairun Nain Nor Aripin, Tengku Amatullah Madeehah Tengku Mohd

**Affiliations:** ^1^ Universiti Sains Islam Malaysia Kuala Lumpur Malaysia; ^2^ Hospital Ampang Ampang Malaysia

**Keywords:** mobile applications, computer-assisted decision making, drug monitoring, pharmacovigilance, adverse drug reactions

## Abstract

**Background:**

Adverse drug reactions (ADRs) cause significant morbidity and mortality. Improved assessment of ADRs to identify the causal relationship, the severity, and the preventability will aid ADRs prevention or reduce patient burden.

**Objective:**

The aim of this study was to develop mobile apps in assisting clinical decision in ADR assessments of causality, severity, and preventability using validated tools. The usability of the apps was assessed.

**Methods:**

We designed mobile apps using validated assessment tools for ADRs. They are the Liverpool ADRs Causality Assessment Tool, Hartwig’s Severity Assessment Scale, and the Modified Schumock and Thronton Preventability Scale. The apps were named “Adverse Drug ReactionCausality,” “Adverse Drug ReactionSeverity,” and “Adverse Drug RxnPreventability.” A survey was conducted using the System Usability Scale (SUS) to assess the usability of the developed apps among health care professionals.

**Results:**

These apps are available for download through Google Play Store for free since January 2015. From the survey, the mean SUS score was 70.9 based on 26 responses from the pediatric ward of Hospital Ampang, Malaysia.

**Conclusions:**

The developed apps received an overall acceptable usability among health care professionals. The usage of these apps will improve detection, assessment, and avoidance of future ADRs. They will also contribute to future research on ADRs, thus increasing drug safety.

## Introduction

Adverse drug reactions (ADRs) cause significant mortality and morbidity in patients [1-5]. The World Health Organization (WHO) defines an ADR as a response to a drug that is noxious and unintended and occurs at doses normally used in man for prophylaxis, diagnosis, or therapy of disease, or for modification of physiological function [6]. Previous studies have shown that ADRs were the cause of 3% of all hospital admissions in the pediatric population and that 10% of children suffer an ADR while in hospital [7]. Similar numbers are seen in adult patients [8]. ADRs have been estimated to cause 3% of all deaths in the general population and up to 5% of deaths in hospitalized patients [4].

Previous studies have shown that the combined use of mobile technology and mobile apps software in health care offer various benefits to many parties including health care professionals, patients, management, and even stakeholders [9-13]. Mobile technology facilitates efficient delivery of services to patients. It also improves quality and effectiveness of services to the benefit of patients [14,15]. However, the use of apps in health care services does not seem as extensive as compared with other services such as social or public services [10,16]. Research in this area is needed to keep up with the increasing number of ADRs and integrate new knowledge with the rapidly advancing technology [9].

According to the WHO, ADR causality assessment is “a method by which it estimates the relationship concerning the agent (which is the drug) and the adverse reactions” [17]. It assesses the causal connection between the drugs and their adverse effects. Assessment of ADRs causality will give an advantage in the ability to classify the relationship, improve scientific evaluation for each individual ADR, and thus enable for an early warning system for clinicians, pharmacists, and health regulators [18-20].

The term severity in ADRs is used to describe the intensity of the adverse drug reaction [21,22]. Similar to causality assessment, severity assessment of ADRs are also crucial in epidemiological studies. The ability to classify the severity of ADRs will provide a mechanism for the health care workers and authorities to identify the problem areas and improve the intervention for patient care that would reduce the burden of ADRs [7,23,24].

Assessment of preventability is important for ADRs as it gives important information to improve prescription practice and enhance patient monitoring [16,24]. Although the assessment of each ADR’s causality, severity, and preventability is crucial to provide important drug safety information, relatively few of these assessments are being performed [7].

This study was designed to develop apps for the aforementioned ADR assessments using validated tools. The apps can be downloaded on a mobile phone or mobile devices, which are then adapted to improve knowledge on ADRs and ultimately drug safety in health care.

## Methods

### Assessment Tool for Adverse Drug Reaction (ADR) Causality, Severity, and Preventability

There is currently no operational tool that has been proven as a gold standard for each ADR assessment of causality, severity, and preventability; therefore, the most widely used or accepted operational tools were selected for the development of our apps. Each of these tools has been validated by previous studies [16,25-29].

The ADR causality app was developed using the Liverpool ADR causality assessment tool [28]. This is a questionnaire-based classification for suspected ADRs using an algorithm built by a multidisciplinary team from the University of Liverpool in 2012. The algorithm classifies the suspected ADRs as definite, probable, possible, or unlikely. Results from a systematic review on assessment of ADR causality showed that the Naranjo algorithm was the most frequently used tool [7]. However, the Liverpool ADR causality assessment tool showed full range of causality category and good interrater reliability (IRR) compared with Naranjo algorithm. Thus, this tool was used in developing the ADR causality app [28].

The ADR severity app was developed based on the Hartwig’s Severity Assessment Scale [24], which is the most commonly used severity tool in ADR studies. It classifies the ADR into mild, moderate, or severe based on level of clinical outcomes [7].

The ADR preventability app was developed using the Modified Schumock and Thornton Preventability Scale [29], which is the most frequently used scale in ADR studies in children [7]. It is a questionnaire on the criteria for determining preventability of ADRs based on clinical circumstances surrounding the ADR. The category of preventability is either definitely preventable, probably preventable, or not preventable.

### Development and Publishing the App Into Google Play Store

The apps were developed using the rapid application development (RAD) model [30]. Using this model, the development processes are divided into three main phases which are preproduction, production, and postproduction.

For the development of the ADR assessment apps, Windows 8.1 by Microsoft was used as the operational system. MIT App Inventor Tool version 2.3.0 [31] was used during the production process and aiStater emulator [32] was then used to provide communication between App Inventor running in the browser and other parts of App Inventor. App Inventor is a free, cloud-based service accessed with a Google account.

After all the production phases were completed, the app was then saved in an APK file and then uploaded into the Google Play Developer Console. Once the ADR assessment app was published in Google Play Store, it could then be downloaded and installed for free by Android OS users.

**Table 1:** Adverse drug reactions (ADRs) assessment tool.

Assessment tool	Reference	App name
Causality assessment	Liverpool Adverse Drug Reaction Causality Assessment Tool [28]	Adverse Drug ReactionCausality
Severity assessment	Hartwig’s Severity Assessment Scale [24]	Adverse Drug ReactionSeverity
Preventability assessment	Modified Schumock and Thronton Preventability Scale [29]	Adverse Drug RxnPreventability

The apps were designed without storage capacity to avoid issues regarding patient confidentiality or personal data. Therefore, information input into the system is not available to anyone. The apps are also accessible for offline use. The details for each ADR app and the references used are shown in Table 1.

### Testing and Measure of App Usability

The System Usability Scale (SUS), a reliable and low-cost usability scale, was used to assess the usability of the ADR app. SUS is a 10-item scale presented with a 5-point Likert scale, which results in an overall score from 0 to 100 that indicates the perceived usability of the interface [33].

A survey was conducted among 26 health care professionals in the pediatric ward of Hospital Ampang, Malaysia, where they were asked to answer the SUS questionnaire. The survey was conducted 10 months after the introduction of the apps among staff at the pediatric ward of Hospital Ampang.

Results of the SUS questionnaire were recorded and normalized using SPSS version 20 (IBM Corp). The mean SUS score and the standard deviation (SD) were then recorded. Products with scores <70 were considered candidates for increased scrutiny, and continued improvement was judged to be marginal at best [34].

## Results

### App Development and Publication

**Figure 1:**
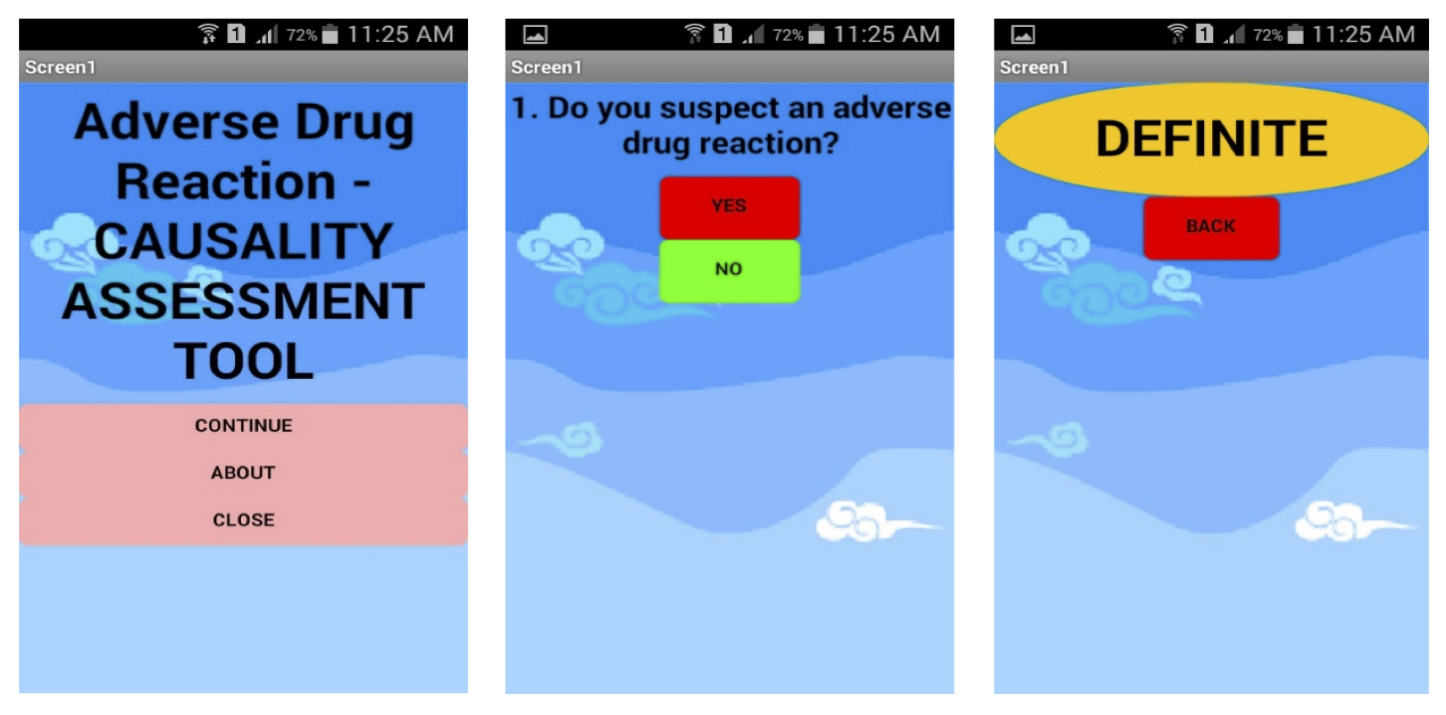
Exemplar of adverse drug reaction (ADR) causality assessment tool screenshot.

**Figure 2:**
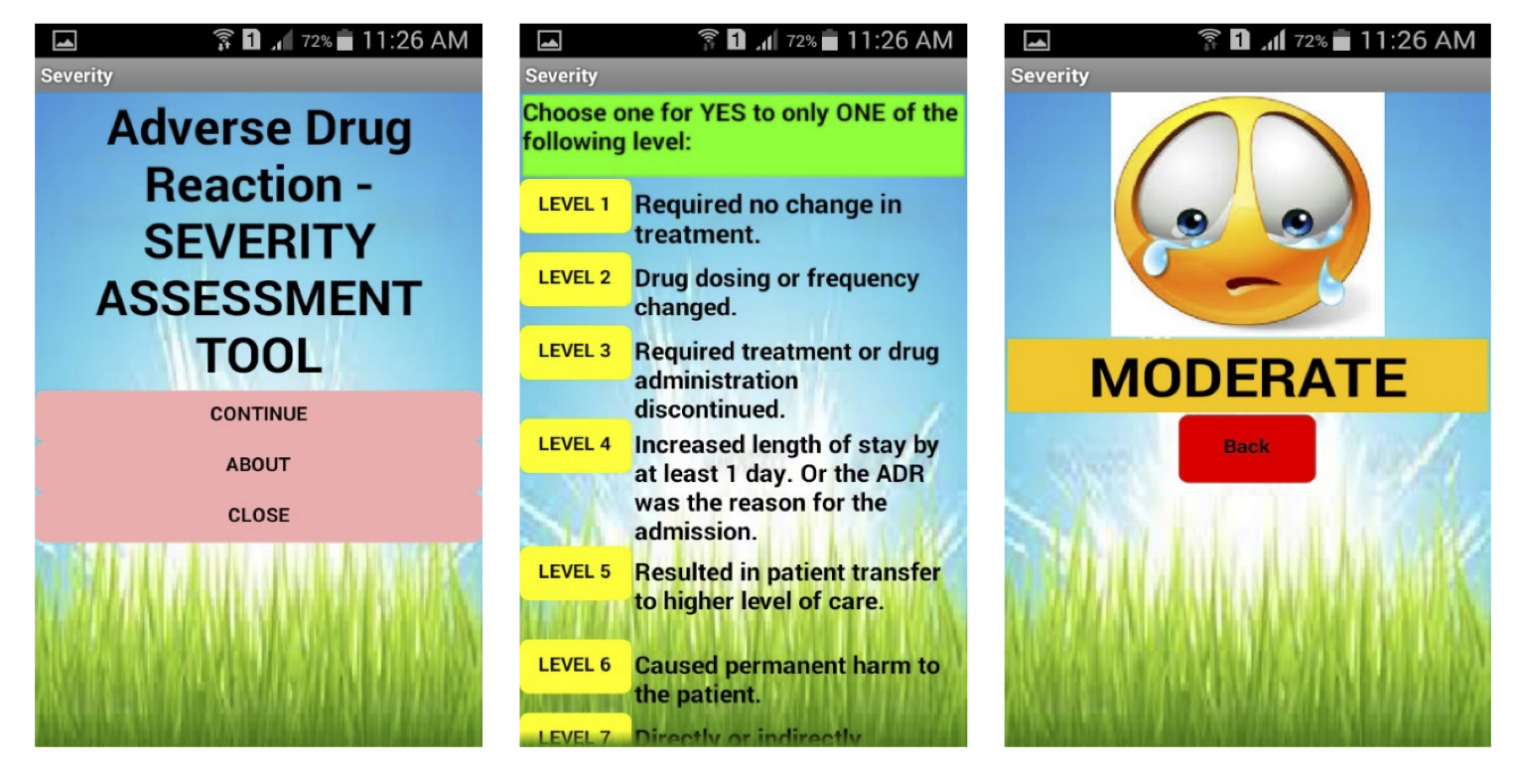
Exemplar of adverse drug reaction (ADR) severity assessment tool screenshot.

**Figure 3:**
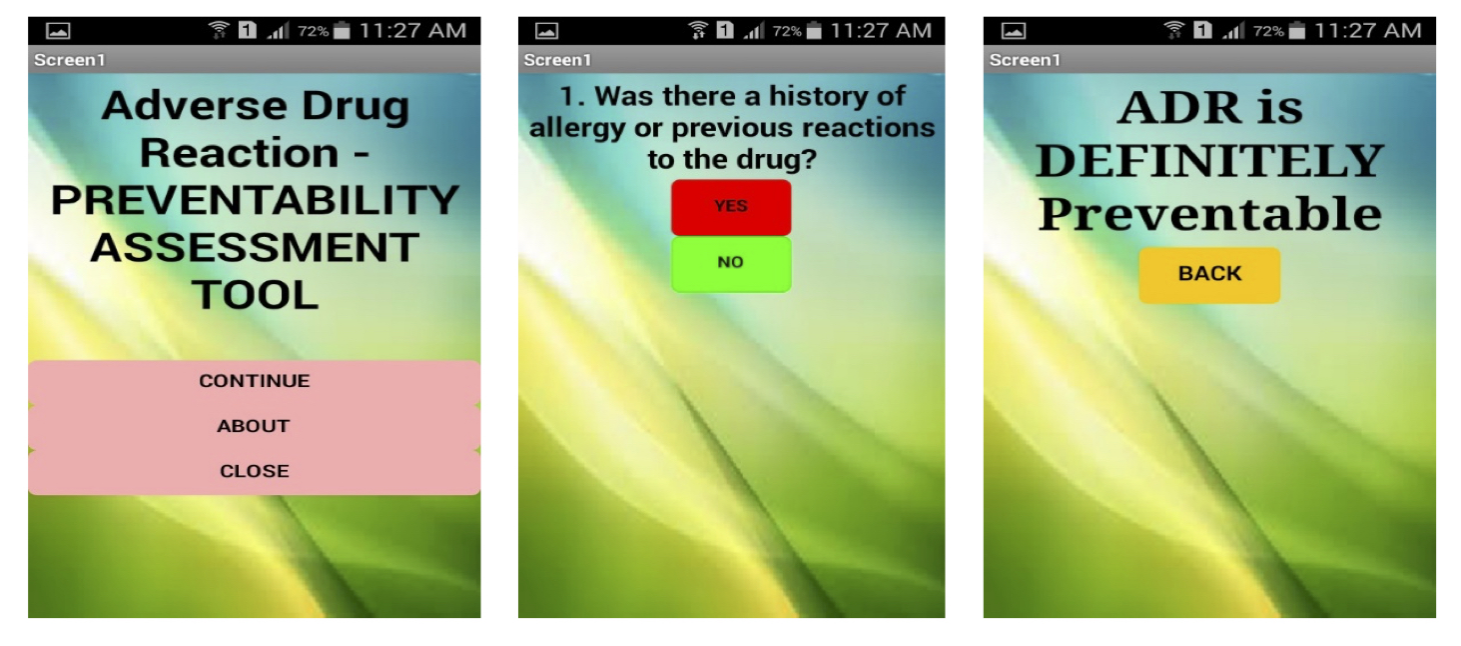
Exemplar of adverse drug reaction (ADR) preventability assessment tool screenshot.

The developed apps were published in Google Play Store on January 22, 2015. All 3 apps were considered to have fulfilled the objective of the development. The apps were freely downloadable from Google Play Store from February 2015. Exemplar screenshots for each app are shown in Figure 1 for causality, Figure 2 for severity, and Figure 3 for preventability.

Up until January 20, 2017, a total of 609 users have downloaded the apps. The total installer, installer by country, and ranking statistics for each ADRs assessment app are shown in Table 2. The highest numbers of downloads were for the causality app followed by the severity and preventability apps. The installers were mainly from India and Malaysia for all the apps.

### App Usability Among Health Care Professionals

Of the 26 health care professionals involved in the survey, 19 (73.1%) of the respondents were physicians, 6 respondents (23.1%) were nurses, and 1 respondent (3.8%) was a pharmacist. The mean SUS score was 70.9 (SD 12.86). The results showed that the SUS score was >70; thus, the app tested is within the acceptable range of usability [34]. Table 3 depicts the responses to the usability-related questions.

**Table 2:** Statistics of adverse drug reaction (ADR) assessment apps from Google Play Store.

Characteristics	Adverse Drug ReactionCausality	Adverse Drug ReactionSeverity	Adverse Drug RxnPrevenrability
Total installers	274	200	135
**Installer by country (%)**	India 77 (28.1)	Malaysia 70 (35)	India 48 (35.5)
	Malaysia 69 (25.0)	India 61 (30.5)	Malaysia 31 (23.0)
	Saudi Arabia 43 (15.6)	Qatar 17 (8.5)	Qatar 16 (11.9)
	South Africa 17 (6.3)	Saudi Arabia 17 (8.5)	Others 40 (29.6)
	United States 17 (6.3)	South Africa 17 (8.5)	
	Others 51 (18.7)	Others 18 (9)	
Average ratings	4.75	5.00	4.50

**Table 3:** Usability questions and summary of responses (N=26).

Answer option	Strongly disagree n (%)	Disagree n (%)	Neutral n (%)	Agree n (%)	Strongly agree n (%)
I think that I would like to use this system frequently	0 (0)	0 (0)	3 (11)	21 (81)	2 (8)
I found the system unnecessarily complex	5 (19)	10 (38)	8 (31)	3 (12)	0 (0)
I thought the system was easy to use	0 (0)	1 (4)	1 (4)	18 (69)	6 (23)
I think that I would need the support of a technical person to be able to use this system	6 (23)	11 (42)	3 (12)	6 (23)	0 (0)
I found the various functions in this system were well integrated	1 (4)	2 (8)	7 (27)	14 (53)	2 (8)
I thought there was too much inconsistency in this system	3 (12)	12 (45)	9 (35)	2 (8)	0 (0)
I would imagine that most people would learn to use this system very quickly	0 (0)	0 (0)	4 (15)	14 (54)	8 (31)
I found the system very cumbersome to use	5 (19)	12 (46)	5 (19)	4 (16)	0 (0)
I felt very confident using the system	0 (0)	0 (0)	7 (27)	16 (61)	3 (12)
I needed to learn a lot of things before I could get going with this system	5 (19)	15 (57)	3 (12)	3 (12)	0 (0)

## Discussion

### Principal Findings

The causality app had the highest number of installers so far. This seems to be similar with previous 102 published ADR studies where causality was the most common assessment conducted in suspected ADR cases [7].

Based on the country of origin, the highest percentage of installers were from India for all of the ADR apps published. India is currently working to strengthen its pharmacovigilance program due to the rapidly growing number of ADR studies in the country [35-38]. We expect that our ADR apps would be able to assist not only Indian researchers and clinical researchers but also any center conducting research on drug safety.

The apps usability among health professionals in the hospital was assessed using SUS, which consisted of 10 alternate statements of positive and negative items rated using a 5-point Likert scale. Our survey results show that the apps developed have a mean SUS of 70.9 (SD 12.86), thus demonstrating acceptable usability.

The health care professionals that used the apps concluded that the apps were convenient and they would choose them over conventional paper-based assessments. Previous studies have found that the use of apps in health care is cost-effective, faster, easier, and more interactive due to factors of mobility, convenience, and involvement of active touching of the screen to perform the assessment [11,39]. The apps are also secure, as they do not store any information from the data inputted into the app [40].

The use of medical apps by health care professionals and researchers, and the numbers of these apps are increasing rapidly. Apps can give additional advantages at the point of care such as in diagnosis, monitoring, reporting, or follow-up of treatment [12]. The increased usage of mobile phone or mobile device apps warrant further studies evaluating their utility and effectiveness on a larger scale.

### Limitations

We have identified a few limitations of the apps. On a basic level, there is no assessment tool universally accepted or described as the gold standard for ADRs either for causality, severity, or preventability. We chose the most widely used and validated algorithms and scales to develop the apps; however, we recognize that not all researchers will agree with the algorithms chosen in development of ADR assessment.

Second, the apps have only been evaluated by health care professionals from the pediatric department in a hospital setting. Further evaluation is necessary to gain more feedback from a wider range of users.

Finally, the aesthetics of the app contents in terms of color, text letters, and pictures have been optimized; however, there is room for improvement to make the apps more attractive.

We are continuously working to update and upgrade the apps. Future research is needed to test the usability of the apps in varying populations and to add several other commonly used algorithms or tools in ADRs assessment. Research to highlight the context and content of the apps should also be designed specifically for health care professionals, researchers, and regulators.

### Conclusions

These ADR assessment apps will aid health care professionals in determining the causality, severity, and preventability of ADRs. This is aimed to contribute toward efforts to reduce the burden of ADRs on patients. The SUS score data showed that the apps have acceptance usability among health care professionals. They will also support future research to enhance overall safety relating to drugs given to patients.
